# Supplementary figures and images for: Prevalence and serotypes of Shiga toxin-producing Escherichia coli (STEC) in dairy cattle from Northern Portugal
Source: PLoS One. 2020 Dec 31;15(12):e0244713. doi: 10.1371/journal.pone.0244713 (PMC7774927; doi:10.1371/journal.pone.0244713)

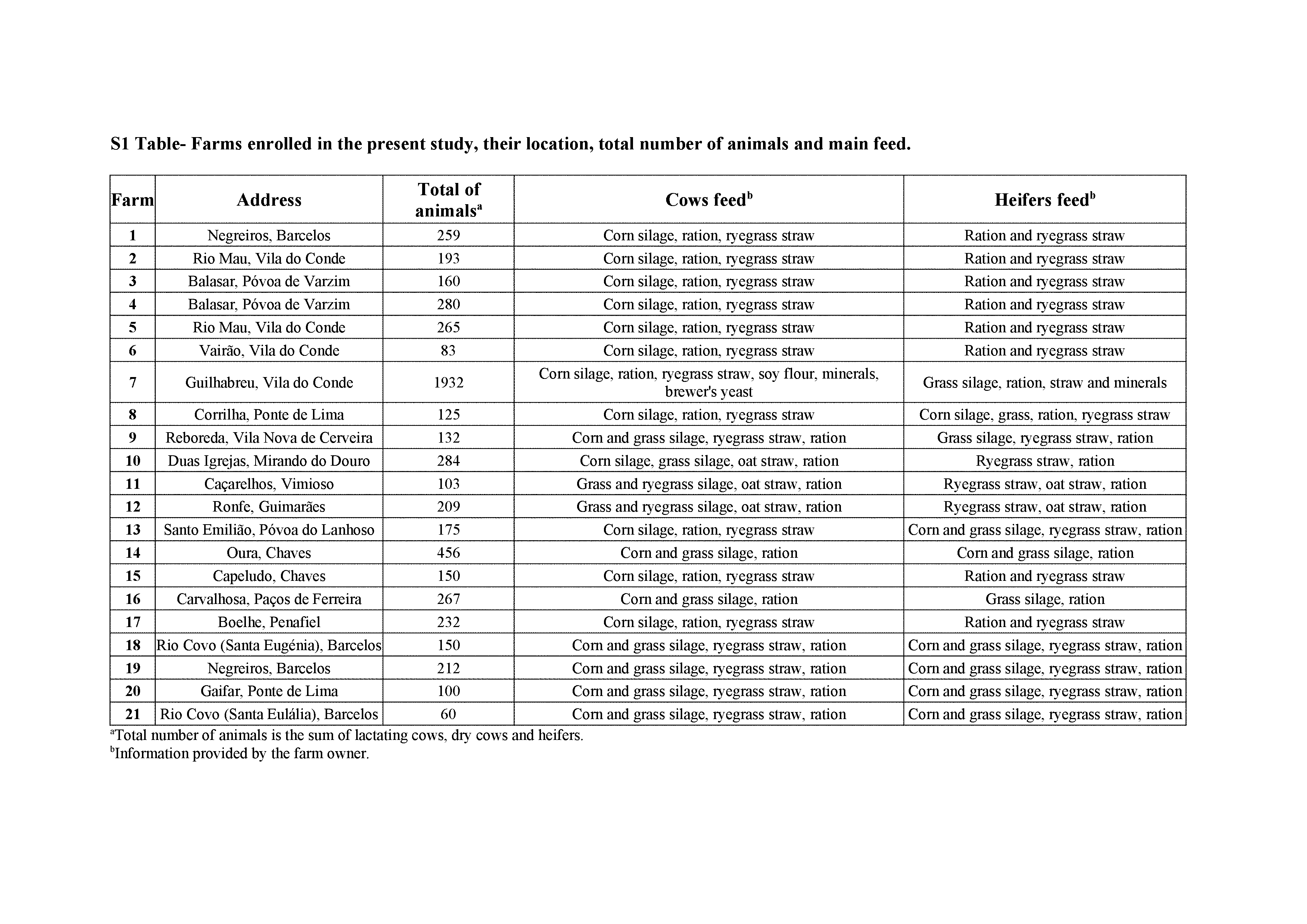

Supplement: S1 Table — aTotal number of animals is the sum of lactating cows, dry cows and heifers. bInformation provided by the farm owner. (TIF) [file pone.0244713.s001.tif]
