# Supplementary material for: Prevalence and serotypes of Shiga toxin-producing Escherichia coli (STEC) in dairy cattle from Northern Portugal
Source: PLoS One. 2020 Dec 31;15(12):e0244713. doi: 10.1371/journal.pone.0244713 (PMC7774927; doi:10.1371/journal.pone.0244713)
Supplement: Attachment — Submitted filename: Paper Review.docx [file pone.0244713.s003.docx]

**Prevalence and Serotypes of Shiga Toxin-Producing Escherichia coli (STEC) in Dairy Cattle from Northern Portugal.**

The reviewer feel that the paper contains valuable scientific research which would benefit people in similar or closely related field. However, the paper would require a bit of some work prior to publication, areas of which are listed below followed by specific areas:

1. There is need for though proof reading to correct on grammar and some tenses.
2. Detailed explanation why there were variations on STEC prevalence in Heifers and cows
3. How did the study consider factors such as season, management systems between farms etc during sample collection? Any adjustments made for these factors among others so as to avoid bias.

**Abstract**

Include what this - stx1 and stx2 stands for at the start then can be followed by use of short form.

The prevalence of STEC in heifers was significantly higher than in lactating cows (include p value)

**Introduction**

51-52 – Did you mean third place in Europe?

78 - Revisit the wording on how you have structured the aim at the start of the sentence.

**Materials and methods**

83-91 – No need to include the names of the persons

93: check on tenses and also review the sentence

98: what criteria was used for deciding to work with farms >50 herd size?

100-101: though mentioned in the abstract the clarity on type of samples collected not shown

105: software for mapping should not be included on the figures section instead should be somewhere on the materials and methods.

111: neighbour?

**Results and discussion**

Information why the STEC prevalence was higher in heifers than milking cow is lacking in this work.

Also, the study needs to show how it took care of animals kept outside compared to those managed indoors. No clear information whether the animals were indoors throughout or were at times given the opportunity to graze outside during warmer months etc?

Worth detailing why the stx1 isolates was more common in adult cows compared to heifers;

214 however, the proportion of isolates harbouring both genes were higher in heifers.

How did the study consider factors such as season? This needs to be detailed in the stud
