# Supplementary material for: Prevalence and serotypes of Shiga toxin-producing Escherichia coli (STEC) in dairy cattle from Northern Portugal
Source: PLoS One. 2020 Dec 31;15(12):e0244713. doi: 10.1371/journal.pone.0244713 (PMC7774927; doi:10.1371/journal.pone.0244713)
Supplement: Attachment — Submitted filename: Response to Reviewers_v2.docx [file pone.0244713.s004.docx]

Please see below for specific responses to reviewers' comments and inquiries. All page numbers refer to the revised manuscript file with tracked changes.

Editor Comments

1. Please ensure that your manuscript meets PLOS ONE's style requirements, including those for file naming. The PLOS ONE style templates can be found at

Authors response: We believe that everything is now in order.

2.We note that [Figure(s) 1] in your submission contain [map/satellite] images which may be copyrighted. All PLOS content is published under the Creative Commons Attribution License (CC BY 4.0), which means that the manuscript, images, and Supporting Information files will be freely available online, and any third party is permitted to access, download, copy, distribute, and use these materials in any way, even commercially, with proper attribution. For these reasons, we cannot publish previously copyrighted maps or satellite images created using proprietary data, such as Google software (Google Maps, Street View, and Earth). For more information, see our copyright guidelines: <http://journals.plos.org/plosone/s/licenses-and-copyright>.You may seek permission from the original copyright holder of Figure(s) [1] to publish the content specifically under the CC BY 4.0 license.

Authors response: Figure 1 (Map of the North of Portugal) was constructed with MicroStation v8 (Bentley Systems, Incorporated) using open data (public domain) from the Portuguese government that have Creative Commons Attribution 4.0 - CC BY 4.0 and are available at <https://dados.gov.pt/pt/datasets/concelhos-de-portugal/> access on 12 February, 2020. This information has now been included in the legend of the figure.

3.We note that you have provided funding information that is not currently declared in your Funding Statement. However, funding information should not appear in the Acknowledgments section or other areas of your manuscript. We will only publish funding information present in the Funding Statement section of the online submission form.

Please remove any funding-related text from the manuscript and let us know how you would like to update your Funding Statement. Currently, your Funding Statement reads as follows:  [Funding statment is presented in the manuscript body]

Please address the following queries:

a)	Please clarify the sources of funding (financial or material support) for your study. List the grants or organizations that supported your study, including funding received from your institution.

b)	State what role the funders took in the study. If the funders had no role in your study, please state: “The funders had no role in study design, data collection and analysis, decision to publish, or preparation of the manuscript.”

c)	If any authors received a salary from any of your funders, please state which authors and which funders.

d)	If you did not receive any funding for this study, please state: “The authors received no specific funding for this work.”

Authors response: Agreed. In fact all the different projects have provided financial (not material) support to the different institutions involved. This is now clearly stated. The Funding and Acknowledgments sections have been updated. It now reads as:

“This study was financially supported by: i) project PhageSTEC (POCI-01-0145-FEDER-029628) funded by FEDER through COMPETE2020 (Programa Operacional Competitividade e Internacionalização) and by National Funds thought FCT (Fundação para a Ciência e a Tecnologia); ii) strategic project UIDB/04469/2020 unit and BioTecNorte operation (NORTE-01-0145-FEDER-000004) funded by FCT under the scope of the European Regional Development Fund (Norte2020 - Programa Operacional Regional do Norte); iii) project PI16/01477 from Plan Estatal de I+D+I 2013-2016, Instituto de Salud Carlos III (ISCIII), Subdirección General de Evaluación y Fomento de la Investigación, Ministerio de Economía y Competitividad (Gobierno de España) and FEDER; and iv) grant ED431C2017/57 from the Consellería de Cultura, Educación e Ordenación Universitaria, (Xunta de Galicia) and FEDER; UIDB/AGR/04033/2020 by National Funds thought FCT. Author IGM acknowledges the Consellería de Cultura, Educación e Ordenación Universitaria, Xunta de Galicia for the individual grant ED481A-2015/149 and and author SCFS acknowledges the FPU programme for the individual grant FPU15/02644 from the Secretaría General de Universidades, Spanish Ministerio de Educación, Cultura y Deporte.

The funders had no role in study design, data collection and analysis, decision to publish, or preparation of the manuscript.”

Reviewer 1

Introduction

• Rephrase line 68 – ‘STEC are found in a wide variety of animal species as a natural gut inhabitant.’

Authors response: The sentence has been reformulated according to the suggestion.

• Lines 69-72: Rephrase to demonstrate that the food is likely contaminated with faeces containing STEC, rather than saliva/blood etc. (e.g. ref: Fairbrother & Nadeau, 2006).

Authors response: Agreed. The sentence was revised to include the suggestion. See lines 72 to 74.

• Line 78-9: ‘investigate the prevalence of STEC in healthy dairy cattle…’. Do you need to add ’and heifers’ as they were referred to separately in the abstract (L29).

Authors response: Agreed. See line 82.

Methods

Methods

• Line 120 – do not need ‘the’ in that sentence.

Authors response: Agreed.

• Line 123 – there isn’t a ‘quantitative’ aspect to the PCR then I would refer to it as presence-absence real time PCR (e.g. PA-PCR)

Authors response: Agreed. Since real time PCR was only used for the evaluating the presence/absence of specific genes, we have removed the reference to “qPCR”. For consistence, we have kept "**real-time PCR"** in the whole text.

• Line 123 – sensitivity of the method would be good to include and any discussion surrounding the potential for inhibition by other components from the enriched faeces or reference the method as being suitable to avoid inhibition by compounds (such as humic acids) that may decrease sensitivity.

Authors response: Agreed. To monitor possible inhibition, we have included an internal amplification control in all real-time PCR runs/tubes. The internal amplification control consisted of a plasmid (pUC19) and the corresponding primers (see Table 1). When inhibition was noticed, samples were diluted 1/10 and retested again. This information has been included in the text (see lines 166 and 174 to 176).

• Line 134 – No controls discussed for the extraction of DNA from enriched faecal slurry. Should include information regarding a positive and negative control for the extraction process as well as the PCR plate set-up, particularly due to the use of an extraction method not designed for faeces.

Authors response: We do understand the reviewer concerns and, in fact, we run artificially inoculated samples periodically as part of our quality/accreditation system. In this particular case, we have previously tested the procedure in enriched faecal that were artificially inoculated. This information has been included in lines 157 to 159.

Results and discussion

• Line 185 – Remove ‘in fact’.

Authors response: Agreed. It has been removed.

• Line 189 ‘in the Michigan’, remove ‘the’.

Authors response: Agreed. It has been removed.

• Line 191-192 – requires different phrasing.

Authors response: Agreed. The sentence was rewritten. Please see lines 218 to 221.

• The section describing stx1 genes as being more common in adults than heifers, while those STEC carrying both genes were more frequently found in heifers requires more discussion. In conjunction with this I think that the section stating that STEC was more commonly isolated from heifers (45% heifers, 16% of adults) – already reported in other studies. So, despite STEC being more frequently isolated from heifers, stx1 was still more common in the adult population? This section reads more like a statement of results and could do with more discussion and tying in with other literature (Lines 203-214). Are there any farm metadata or other studies that could provide insight?

Authors response: The reviewer makes a relevant comment, as we need to clarify if we refer to absolute number of isolates or to the proportion (%) of isolation with a specific profile. This information was not clear, so we have revised the text to clarify this. Please see lines 251 to 259.

• Line 228- ‘well established’.

Authors response: Agreed.

• Line 228 – missing references.

Authors response: Agreed. We added the missing references Sandhu & Gyles (2002) and Karmali *et al.* (2003) at line 274.

• Line 243 – ‘establishing’

Authors response: Agreed.

• Line 245 – ‘foodborne’

Authors response: Agreed.

• Higher diversity of serotypes from heifers (55) compared to lactating (34 serotypes) but no discussion.

Authors response: Agreed. We have noticed some faults and we revised this information. Please see lines 301 to 305.

• In text it is stated that 29/74 (line 260) but 31 are indicated in bold (Table 2).

Authors response: Agreed. These were in fact faults that we have now corrected.

• More could be done to achieve the objective of evaluating the serotype diversity (line 266). Did some farms/areas have higher diversity than others? Do certain serotypes appear to out compete others? Is it possible to run Simpson/Shannon diversity on these data? Did the farms with high or low diversity have high cattle numbers or an older cattle cohort?

Authors response: Agreed. We have estimated the diversity index of our population of STEC isolates. However, since the number of samples collected per farmer changes significantly (as we have sampled a proportion of animals according to the farm size), we did this estimation for the isolates taken from milking cows and from heifers. This way we can evaluate the diversity according to animals’ age. Please see line 301 to 305.

Conclusion

• Did not mention that Portugal does not have a surveillance system in place for monitoring STEC/VTEC infections like most EU/EEA Member States – could be worth mentioning in the conclusion to make a case for increased surveillance?

Authors response: Agreed. Please see lines 340 and 342.

Reviewer #2: The reviewer feel that the paper contains valuable scientific research which would benefit people in similar or closely related field. However, the paper would require a bit of some work prior to publication, areas of which are listed below followed by specific areas:

1. There is need for though proof reading to correct on grammar and some tenses.

Authors response: Agreed. The paper has been revised accordingly.

2. Detailed explanation why there were variations on STEC prevalence in Heifers and cows

Authors response: Agreed. This information has been added now. Please see line 239 to 247.

3. How did the study consider factors such as season, management systems between farms etc during sample collection? Any adjustments made for these factors among others so as to avoid bias.

Authors response: We do understand the reviewer comments; however, all farms have a similar management system and sampling was schedule according to the farmers’ availability. So, we cannot draws any conclusions on the seasonality or the management systems.

Abstract

Include what this - stx1 and stx2 stands for at the start then can be followed by use of short form.

Authors response: Agreed. This has been included.

The prevalence of STEC in heifers was significantly higher than in lactating cows (include p value)

Authors response: Agreed.

Introduction

51-52 – Did you mean third place in Europe?

Authors response: Agreed. See lines 50 and 51.

78 - Revisit the wording on how you have structured the aim at the start of the sentence.

Authors response: Agreed. See lines 81 and 83.

Materials and methods

83-91 – No need to include the names of the persons

Authors response: Agreed. The names of persons have been removed from sentence.

93: check on tenses and also review the sentence

Authors response: Agreed. The sentence was revised. Please see lines 97 to 100.

98: what criteria was used for deciding to work with farms >50 herd size?

Authors response: We have chosen to sample heifers and lactating cows on the same farm and farms with small herds usually do not have heifers. Also, very small sample sizes could prejudice the prevalence estimation per herd.

100-101: though mentioned in the abstract the clarity on type of samples collected not shown

Authors response: Agreed. We have revised the text. See line 104 and 105.

105: software for mapping should not be included on the figures section instead should be somewhere on the materials and methods.

Authors response: Agreed. Reference to the software was removed from the figure and included in the M&M (lines 107 to109).

111: neighbour?

Authors response: 'neighbour' was replaced by ‘neighbouring country’.

Results and discussion

Information why the STEC prevalence was higher in heifers than milking cow is lacking in this work.

Authors response: We have now included some discussion on this matter as also recommended by reviewer 1. Please see lines 239 to 246.

Also, the study needs to show how it took care of animals kept outside compared to those managed indoors. No clear information whether the animals were indoors throughout or were at times given the opportunity to graze outside during warmer months etc?

Authors response: We understand the reviewer concern. In fact, all sampled farms kept animals indoors, but in open stables. At times, animals were given the opportunity to graze outside. This information has now been included – lines 108 to 109.

Worth detailing why the stx1 isolates was more common in adult cows compared to heifers;

214 however, the proportion of isolates harbouring both genes were higher in heifers.

How did the study consider factors such as season? This needs to be detailed in the study.

Authors response: We do understand the reviewer concerns; however, as stated above to reviewer 1, sampling was schedule according to the farmers’ availability. So, we cannot draws any conclusions on the seasonality. This should be taken as an average prevalence for this particular period. Nonetheless, to clearly state this limitation, we have revised the text to include this information. Please see lines 239 to 247.
